# Aging Aggravates Nitrate-Mediated ROS/RNS Changes

**DOI:** 10.1155/2014/376515

**Published:** 2014-03-23

**Authors:** Qian Fan, Lifen Chen, Shujuan Cheng, Fang Li, Wayne Bond Lau, Le Feng Wang, Jing Hua Liu

**Affiliations:** ^1^Department of Cardiology, Beijing An Zhen Hospital, Capital Medical University, Beijing Institute of Heart, Lung and Blood Vessel Disease, Beijing 100029, China; ^2^Department of Neurology, The Second Affiliated Hospital of Chong Qing Medical University, Chong Qing, China; ^3^Department of Emergency Medicine, Thomas Jefferson University, Philadelphia, PA 19107, USA; ^4^Heart Center, Beijing Chaoyang Hospital-Affiliate of Beijing Capital Medical University, 8 Gongtinan Road, Beijing 100020, China

## Abstract

Nitrates are the most frequently prescribed and utilized drugs worldwide. The elderly are a major population receiving nitrate therapy. Both nitrates and aging can increase in vivo reactive oxygen species (ROS) and reactive nitrogen species (RNS). To date, the effects of aging upon nitrate-induced ROS/RNS alteration are unknown. The present study tested the effects of aging upon nitrate-induced ROS/RNS alteration in vivo. 32 adults and 43 elderly unstable angina (UA) patients were subjected to 48 hours of isosorbide dinitrate intravenous injection (50 **μ**g/minutes) in this clinical study. Blood samples were obtained at baseline and conclusion. Outcome measures of oxidative stress included plasma malondialdehyde (MDA), myeloperoxidase (MPO), and reduced glutathione (GSH). Plasma concentrations of NO*x* and nitrotyrosine served as markers of RNS. Because of the significant differences in basic clinical characters between adults and the elderly, we designed an additional experiment determining ROS/RNS stress in rat cardiac tissue. Additionally, rat thoracic aortic NOS activity served as a marker indicating endothelial function. Our study demonstrated that nitrate therapy significantly increased in vivo ROS/RNS stress in the elderly compared to adult patients, confirmed by animal data. Decreased NOS activity was observed in old rats. Taken together, the present study's data suggests a synergism between nitrate treatment and the aging process.

## 1. Introduction

Rapid growth of the world's geriatric population has increased awareness of age-related cardiovascular diseases. Cardiovascular diseases are responsible for the majority of elderly mortality. 80 percent of patients with ischemic heart disease are ≥65 years old [1]. Organic nitrates have been employed in the treatment of ischemic heart disease for more than a century and remain the most frequently prescribed and utilized medications for treating the ischemic heart disease population worldwide, of which the elderly are a major constituent.

The process of aging is complex. Senescent pathophysiology arises from various factors through multiple mechanisms. Harman proposed the free radical theory of aging in the 1950s, expanding to implicate mitochondrial production of reactive oxygen species in the 1970s [2]. Per this theory, enhanced and unopposed metabolism-driven oxidative stress plays a major role in diverse chronic age-related disorders [3, 4]. In the free-radical theory of aging, organisms age because their cells accumulate free radical damage over time. In our previous study, aging resulted in significantly increased reactive oxygen species (ROS) and reactive nitrogen species (RNS) after myocardial infarction [5, 6]. Nitrate therapy also augments ROS and RNS production. In 1995, Munzel et al. [7] demonstrated that in vivo nitrate use was associated with an endothelial-dependent production of superoxide anion, an important mechanistic development in the understanding of nitrate tolerance. Munzel's work was further supported by the association of reduced superoxide anion production with inclusion of a nitrate-free period in vivo [8]. A randomized controlled trial (RCT) of patients undergoing elective coronary artery bypass grafting subjected to preoperative intravenous nitroglycerin (GTN) confirmed increased superoxide generation in internal mammary artery samples after nitrate treatment [9].

Substantial evidence supports oxidative stress as one of the major etiologies of myocardial injury. Numerous experiments have demonstrated markedly increased superoxide (O_2_^−^) generation from ischemic/reperfused endothelial cells and increased neutrophil activation in postischemic myocardial tissue. O_2_^−^ further dismutates to H_2_O_2_ and ^*∙*^OH, the latter highly toxic to biological tissues, causing significant myocardial necrosis and apoptosis. Additionally, accumulating evidence indicates RNS, such as peroxynitrite (ONOO^−^), play vital roles in reperfusion-induced myocardial apoptosis [10]. The deleterious effects of RNS are further exacerbated by interaction with increased ambient ROS. Most studies investigating nitrate use have employed young animals. Although it is well known that both aging and nitrates increase in vivo ROS/RNS production; heretofore, the effects of nitrate administration in the aged population remain unknown.

The present study determined whether nitrates increase in vivo ROS/RNS concentrations in a clinical trial. Limitations of our study included clinical and demographic characteristic (blood pressure, diabetes, and past medical history) discrepancies between middle-aged and elderly patients and inability to obtain cardiac tissue samples from enrolled participants. A carefully controlled experiment upon rats was therefore included in the present study.

## 2. Materials and Methods

The clinical trial was carried out in accordance with the Declaration of Helsinki (2000) of the World Medical Association. The study protocol was approved by the institutional ethics committee of the Beijing Anzhen Hospital-Affiliate of Capital Medical University. After full disclosure of the study's purpose, nature, and inherent risks of participation, all subjects gave written informed consent prior to enrollment.

### 2.1. Inclusion and Exclusion Criteria of Acute Myocardial Infarction Patients

Unstable angina (UA) patients met inclusion criteria if the following conditions were true: (1) presence of typical angina, (2) presence of electrocardiographic S-T segment changes (representative of cardiac injury) during clinical chest pain, or (3) presence of the significant coronary stenosis (≥75%) diagnosed by either computed tomography coronary angiography (CTCA) or coronary angiography.

Exclusion criteria for this study included (1) increasing myocardial necrotic marker (cTnI or CKMB) levels; (2) cardiogenic shock; (3) occlusion or severe stenosis of the left main coronary artery; (4) previous myocardial infarction; (5) major infection or surgery within the past 2 weeks prior to presentation.

### 2.2. Coronary Angiography and Clinical Experimental Design

In present study, isosorbide dinitrate was used in the clinical trial and nitroglycerin was used in the animal experiment. Both of them are nitrates. Nitrates correct the imbalance between the flow of blood and oxygen to the heart and the work that the heart must do by dilating (expanding) the arteries and veins in the body. Dilation of the veins reduces the amount of blood that returns to the heart that must be pumped. Dilation of the arteries lowers the pressure in the arteries against which the heart must pump. As a consequence of both effects, the heart works less and requires less blood and oxygen.

All UA patients were given standard UA treatment protocol. Patients were divided into two groups: (1) adult patients (<65 years) and (2) elderly patients (≥65 years).

All patients received intravenous isosorbide dinitrate (50 *μ*g/minute) treatment. Blood samples were obtained before and after 48 hours of nitrate treatment.

### 2.3. Animal Experiment Protocol

The study was approved by the institutional ethics committee and was in accordance with the United States National Institutes of Health guidelines. Male Sprague-Dawley rats were anesthetized with sodium pentobarbital. The rat model was described previously [5, 8, 11]. In brief, male Sprague-Dawley rats (aged either 8 weeks (young) or 24 months (old)) were anesthetized with sodium pentobarbital (50 mg/kg body weight) intravenously. An intratracheal tube was inserted via midline midline incision. All rats were given intermittent positive-pressure ventilation with oxygen-enriched room air via Harvard small animal respirator (Harvard Apparatus, South Natick, MA). A transfusion needle was inserted into the caudal vein for supplemental pentobarbital injection to maintain anesthesia and for drug administration. Rats (8 weeks or 24 months old) were divided into the following four groups: (1) sham young; (2) sham old; (3) young; and (4) old (*n* = 12 each). Vehicle (1 mL/kg/h) or nitroglycerin (60 *μ*g/kg/h) [8] was continuously infused for 12 hours. Sham rats received vehicle solution only. After 12 hours of vehicle or nitroglycerin infusion, animals were sacrificed. Cardiectomies were performed.

### 2.4. Quantitative Evaluation of Reactive Oxygen Species (ROS) Level in Humans and Rats

To determine the effects of aging upon in vivo ROS, malondialdehyde (MDA), myeloperoxidase (MPO), and reduced glutathione (GSH) served as oxidative stress markers.

Blood samples were drawn from UA patients after 0 and 48  hours of injection. Blood samples were immediately centrifuged at 10000 RPM for 1 minute at 4°C. Supernatant was collected and stored at −80°C until measurement. Plasma malondialdehyde (MDA), myeloperoxidase (MPO), and reduced glutathione (GSH) concentrations were detected by commercially available kits, as reported previously [11].

Plasma MDA, MPO, and reduced GSH concentrations do not directly reflect oxidative stress within cardiac tissue. Therefore, MDA, MPO, and reduced GSH concentrations in rat cardiac tissue were measured. After 12 hours injection or either nitrate or vehicle, left ventricular samples were homogenized and centrifuged for 30 minutes at 10000 PRM at 4°C. Supernatant protein concentrations were measured by the bicinchoninic acid method. MDA, MPO, and reduced GSH concentrations were determined.

### 2.5. Quantitative Evaluation of Reactive Nitrogen Species (RNS) Level in Humans and Rats

NO*x* (nitrite and nitrate, the stable metabolites of NO) quantity in supernatants was determined via Griess reaction utilizing a NO*x* concentration assay kit (R and D Systems Inc., Minneapolis, MN). Rat cardiac tissues were harvested and similarly processed as described above.

Nitrotyrosine is the accepted footprint of in vivo ONOO^−^ formation. Nitrotyrosine concentration of both rat cardiac tissue homogenate and patient plasma was determined via ELISA kit (Cell Sciences Inc., Canton, MA, USA), as previously described, reported as nanomoles of nitrotyrosine/gram of tissue protein homogenate or nanomoles of nitrotyrosine/liter plasma.

### 2.6. The Assay of NOS Activity in Rat Thoracic Aorta

Under physiological conditions, rat thoracic aortic NOS activity indirectly reflects vascular eNOS concentrations and endothelial function. Therefore, in the present study, NOS activity in rat thoracic aorta served as a marker of endothelial function. After 12 hours of nitrate administration, rat thoracic aortae were isolated and harvested. Samples were homogenized and centrifuged for 30 minutes at 12,000 g at 4°C. Supernatant protein concentrations were measured by the bicinchoninic acid method. Methods determining NOS activity have been described previously.

### 2.7. Statistical Analysis

All values are presented as means ± SEM. All biochemical assays were performed in duplicate and averaged. Data were subjected to ANOVA, followed by Bonferroni correction for post hoc Student's *t*-tests. All statistics were calculated utilizing Graphpad Prism 5.0. *P* values <0.05 were considered statistically significant.

## 3. Results

### 3.1. Patient Population Demographics and Characteristics

**Figure 1:**
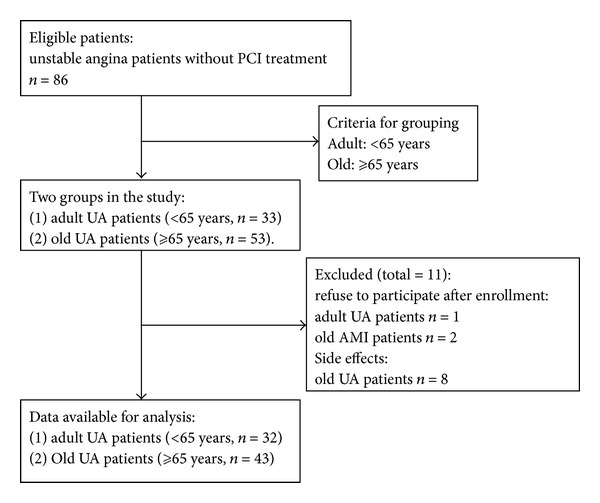
The grouping of clinical trial.

33-adult UA patients and 53 elderly UA patients were enrolled in the clinical trial. Of 86 patients, 1 adult and 2 elderly patients refused participation after enrollment and 8 elderly patients discontinued participation due to side effects (Figure 1).  Table 1 lists all demographic data, baseline statistics, cardiovascular risk profile, and medication profiles of patients. The major differences between the adult and elderly patient populations involved gender distribution and past medical history.

### 3.2. Aging Increased Oxidative Stress in Human Plasma after 48 Hours Nitrate Treatment

**Figure 2:**
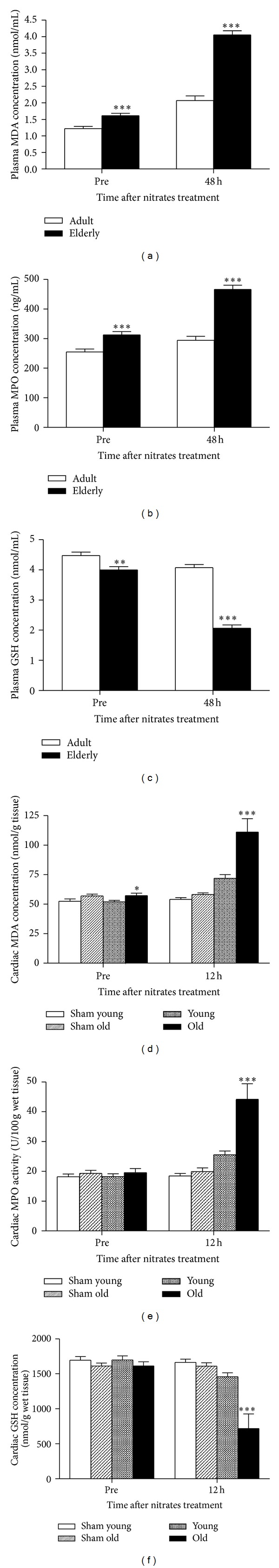
Aging was associated with increased ROS (MDA and MPO) and decreased reduced glutathione (GSH) after nitrates use. (a) Plasma malondialdehyde (MDA) concentration was higher in old patients than in adult patients; (b) plasma-reduced glutathione (GSH) concentration was lower in old patients than in adult patients; and (c) plasma myeloperoxidase (MPO) concentration was greater in elderly than adult patients. (d), (e), and (f) indicated aging was associated with increased ROS (MDA and MPO) and decreased reduced GSH in rat cardiac tissue after 12 hours nitrates use. Adult indicates adult group, and elderly indicates elderly group. Totals for the following groups: 32 adult patients; 43 elderly patients. Sham indicates sham group; young and old indicate rats receiving nitrates treatment. In animal experiment, *n* = 12 per group. Data are expressed as mean ± SEM. ***P < 0.001 versus adult group. *n* = 12 per group. Data are expressed as mean ± SEM. *P < 0.05, **P < 0.001 versus young group.

To determine the effects of aging upon plasma ROS levels after nitrate injection, plasma levels of MDA, MPO, and reduced GSH (markers reflecting myocardial oxidative stress) at two distinct time points (before and after 48 hours nitrate treatment) were determined. Plasma MDA and MPO concentration in elderly UA patients were significantly increased before and after nitrate administration compared to adult patients (Figures 2(a) and 2(b)). Nitrates increased plasma MDA by 140% in the elderly group. Nitrates increased plasma MDA by 60% in the adult group (Figure 2(a)). Nitrates increased plasma MPO by 50% in the elderly group, compared to 20% in the adult group.

Glutathione is one of the most important physiologic self-generating antioxidants. To indirectly evaluate in vivo oxidative stress burden, we determined the plasma concentrations of reduced-form GSH. In consistent trend with plasma MDA and MPO data, elderly patients manifested reduced GSH concentrations compared to adult patients, which further decreased after nitrate administration (Figure 2(c), 48% reduction of elderly plasma GSH after nitrates, compared to 9% reduction in adult patients).

### 3.3. Aging Increased Oxidative Stress in Rat Cardiac Tissue with 12-Hour Nitrate Treatment

Furthermore, in vivo study results confirm the results from clinical trial. Nitrate administration significantly increased rat myocardial MDA and MPO concentrations and decreased myocardial reduced-GSH level in the old group (Figures 2(d), 2(e), and 2(f)).

### 3.4. Aging Increased Plasma NO*x* or Nitrotyrosine Concentrations in Humans after 48-Hour Nitrate Treatment

**Figure 3:**
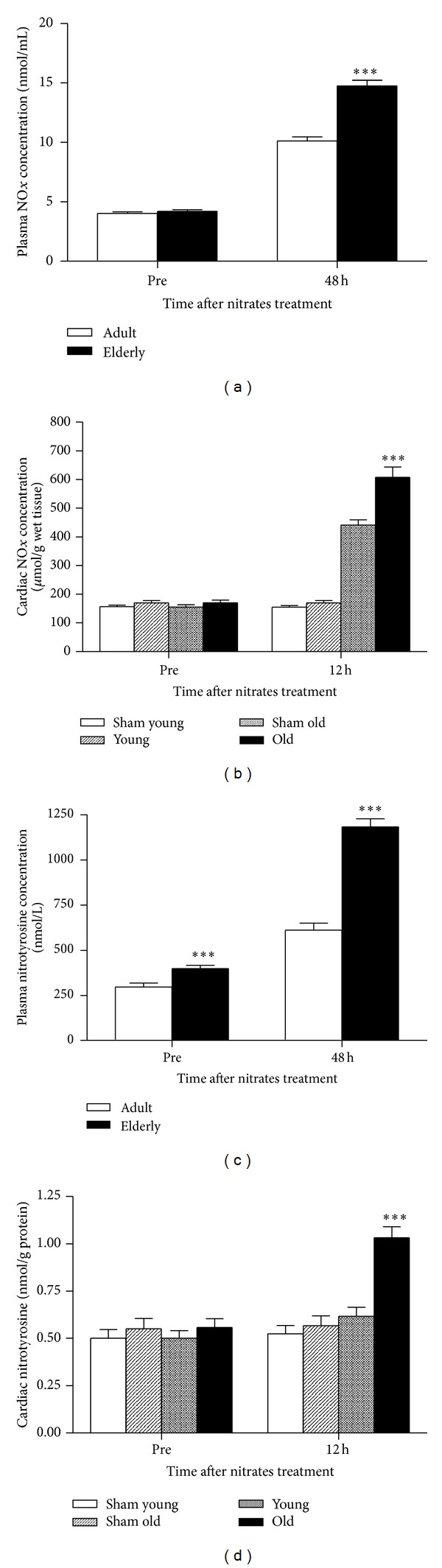
The effects of aging on nitrates-induced NO*x* and nitrotyrosine content change in patients plasma and rats cardiac tissue. (a) indicated the effects of aging on patients plasma NO*x* content; (b) rats cardiac tissue NO*x* content; (c) patients plasma nitrotyrosine concentration; and (d) rats cardiac tissue nitrotyrosine concentration. Nitrates were used in patients for 48 h and in rats for 12 h. Sham indicates sham group; young and old indicate rats receiving nitrates treatment. 32 adult patients and 43 elderly patients were included in the trial. In animal experiment, *n* = 12 per group. Data are expressed as mean ± SEM. ***P < 0.001 versus young/adult group.

RNS (reactive nitrogen species) play a critical pathogenic role in mediating myocardial injury. NO reacts with superoxide (whose production is increased with aging) to form the toxic molecule peroxynitrite (ONOO^−^), a strongly nitrating and oxidizing agent. ONOO^−^ substantially induces myocardial apoptosis. We determined both plasma NO*x* and nitrotyrosine concentrations in the present study. There were no significant differences in plasma NO*x* concentration between adult and elderly patients before nitrate administration. Baseline plasma nitrotyrosine concentration is significantly greater in the elderly than adult population. 48 hours of nitrate injection significantly increased plasma NO*x* (250% in the elderly compared to 150% in the adult patients) and nitrotyrosine (210% in the elderly compared to 105% in the adult patients) concentrations (Figures 3(a) and 3(c)).

### 3.5. Aging Increased Cardiac NO*x* and Nitrotyrosine Concentrations in Rats after 12-Hour Nitrate Treatment

To confirm the effects of aging upon RNS formation, we determined NO*x* and nitrotyrosine concentrations in a rat model after nitrate administration. As shown in Figures 3(b) and 3(d), 12 hours of nitrate infusion significantly increased cardiac NO*x* and nitrotyrosine concentrations in both the young and old rat groups. In the elderly group, NO*x* concentrations increased 255%, compared to 170% in the young group. Similarly, cardiac nitrotyrosine concentrations increased 78% in the old rats, compared to 22% in the young rats.

### 3.6. Aging Increased NOS Activity in Rat Thoracic Aorta after 12-Hour Nitrate Treatment

**Figure 4:**
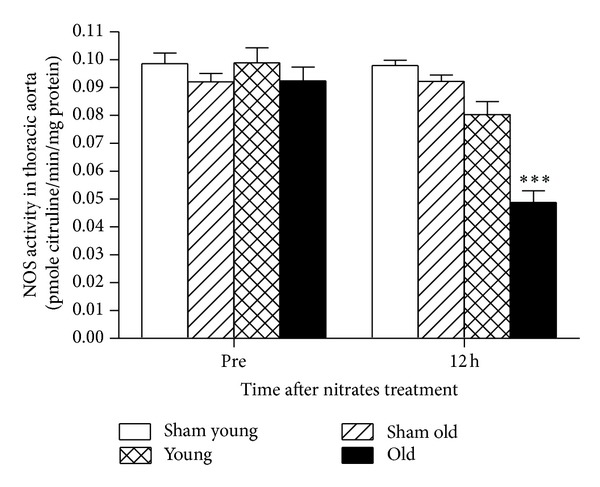
In rats experiment, 12 h nitroglycerin use resulted in the depressing of NOS activity in aorta. Sham indicates sham group; young and old indicate rats receiving nitrates treatment. Data are expressed as mean ± SEM. ***P < 0.001 versus young group.

Both eNOS activity and nitric oxide bioavailability are vital markers indicating endothelial function. Rat thoracic aorta NOS activity served as an indirect index of eNOS activity and endothelial function. As shown in Figure 4, 12 hours of nitrate infusion significantly decreased NOS activity in both young and old rat thoracic aortae. 45% decreased NOS activity was observed in the elderly group, compared to 18% decrease in the young group, suggesting that nitrates induced more severe endothelial dysfunction in the elderly state.

## 4. Discussion

Several novel observations have been made in the present study. Firstly, we demonstrate for the first time with clinical data that nitrate treatment may increase oxidative/nitrative stress burden in the elderly. To our knowledge, previous investigations demonstrating nitrates increased ROS/RNS post I/R involved all young animals, heretofore unconfirmed in human studies. Secondly, although endothelial function depression has been observed after nitrate administration, our results suggest that aging exacerbated such effects.

Recent evidence suggests that the mitochondrial respiratory chain is the primary source of nitrate-induced vascular O_2_^−^ overproduction, leading to subsequent activation of vascular nicotinamide adenine dinucleotide phosphate (NADPH) oxidase, and which mediates the majority of nitrate tolerance and endothelial dysfunction [12]. The superoxide anion is normally scavenged by various intracellular and extracellular mechanisms. Superoxide excess may overcome these compensatory mechanisms and rapidly react with NO (derived in nitrate-dependent fashion) to form toxic peroxynitrite [8, 13]. While the current study confirms the above principles, we provide data suggesting aging may augment nitrate-induced superoxide/peroxynitrite generation. Previously, we demonstrate that aging exacerbated reperfusion-induced myocardial injury [6], particularly apoptosis. It is widely accepted that superoxide anion/peroxynitrite exert severely deleterious myocardial effects. It is possible that nitrate-induced ROS/RNS alteration is an important cause of aging-related myocardial injury.

As a major predictor of cardiac events in patients with coronary artery disease and heart failure, endothelial dysfunction may be the pathologic sequelae of nitrate [14, 15]. Endothelial dysfunction generally reflects reduced nitric oxide bioavailability, due to increased oxidative/nitration stress. Aging is an independent risk factor of cardiovascular diseases such as myocardial infarction and heart failure. Nitrate administration increased in vivo RNS and ROS production, furthermore decreasing nitric oxide bioavailability. The present study indirectly demonstrated aging significantly exacerbated nitrate-induced endothelial dysfunction, both in human and animals. Our results give insight to the contribution of aging to cardiovascular injury.

In summary, the elderly is a population treated extensively by nitrates. However, the effects of aging on nitrates use seem to be neglected. With supporting clinical human data and experimental animal data, the present study suggests a deleterious “synergism” between aging and nitrate treatment upon cardiovascular injury. Aging increases ROS/RNS after nitrate treatment, which may result in more severe myocardial injury. The current study's data warrants further studies investigating the use of nitrates in the elderly, with caveats regarding its judicious employment.

## Figures and Tables

**Table 1:** Baseline Characteristics of the Study Population (mean ± SD).

	MI adult (*n* = 34)	MI elderly (*n* = 45)	*P*
Age, y	50.1 ± 6.6	73.7 ± 4.8	<0.001
Sex, M/F	28/4	24/19	<0.01
HBP/total	23/32	35/43	NS
Dyslipidemia/total	21/32	34/43	NS
Diabetes/total	20/32	29/43	NS
Smoker/total	24/32	29/43	NS
Past drug treatment (*n*/total)			
Statins	17/32	30/43	NS
Calcium channel blocker	9/32	18/43	NS
ACEI	15/32	31/43	<0.05
ß-Adrenoceptor blocker	8/32	26/43	<0.01
Diuretic	5/32	20/43	<0.01
Drug treatment during the study (*n*/total)			
Statins	32/32	43/43	NS
Calcium channel blocker	19/32	32/43	NS
ACEI	17/32	27/43	NS
ß-Adrenoceptor blocker	21/32	27/43	NS
nitrates	32/32	43/43	NS
Mean dosage of nitrates (*μ*g/min)	50	50	NS

HBP indicates high blood pressure; ACEI: angiotensin-converting enzyme inhibitor; y: year; M: male; F: female.
